# Supplementary material for: Decision on optimal airflow regulation solution set based on heat and airflow coupling characteristics of mine airflow in time series
Source: PLoS One. 2025 May 7;20(5):e0320326. doi: 10.1371/journal.pone.0320326 (PMC12058199; doi:10.1371/journal.pone.0320326)
Supplement: Attachment — Submitted filename: Response to Reviewers.docx [file pone.0320326.s003.docx]

**Reviewer Comments & Revision Explanation**

Dear Editors and Reviewers,

Thank you for your thoughtful review of my manuscript. I apologize for the delay in submitting this revised version. During the revision process, I have conducted a detailed collection and secondary utilization of data, particularly focusing on the numerical simulations and practical application calculations that were highlighted in the reviewer comments. This process took longer than anticipated. I kindly request that you review the revised manuscript, and I look forward to your valuable feedback.

**I. Revisions Regarding Journal Requirements**

Thank you for your diligent work and thorough review of my manuscript. This is my first submission to PLOS ONE, and I acknowledge that there were several aspects of my manuscript that did not fully comply with the journal’s requirements. I have carefully reviewed the journal guidelines and have made revisions to both the formatting and content of the manuscript to meet the necessary standards. The revised version of the manuscript, with all adjustments, is included in the resubmitted files for your review.

I greatly appreciate the time and effort you have dedicated to reviewing my work, and I look forward to your further feedback.

Q_1_: Ensure that the manuscript meets PLOS ONE's style requirements.

A_1_: I have carefully reviewed the PLOS ONE style guidelines and ensured that the manuscript meets all formatting requirements. The manuscript has been reformatted according to the templates provided in the links:

Main Body Formatting: The manuscript has been adjusted to align with the style requirements outlined in the sample for the main body.

Title and Author Affiliations: The title page and author affiliations have been formatted according to the provided template.

I have attached the revised manuscript with the appropriate formatting.

Q_2_: Ensure that the code is shared in a way that follows best practice and facilitates reproducibility and reuse.

A_2_: The methods described in the manuscript utilize Matlab's built-in functions and toolboxes for model solving and optimization, and no custom, author-generated code was developed for this study. As a result, there are no specific code contributions that require sharing under PLOS ONE's guidelines.

However, if further clarification is needed or if any other materials are required to ensure reproducibility, I am happy to provide additional information upon request.

Q_3_: Please include your updated Competing Interests statement in your cover letter.

A_3_: I confirm that the statement in the Competing Interests section does not alter our adherence to all PLOS ONE policies on sharing data and materials. We fully commit to sharing the data and materials in accordance with PLOS ONE's policies. If there are any restrictions on sharing, they will be clearly stated in the manuscript.

The updated Competing Interests statement is as follows:

“The authors declare that they have no competing interests exist.”

I have included this updated statement in the cover letter, as requested.

Q_4_: Confirm at this time whether or not your submission contains all raw data required to replicate the results of your study.

A_4_: I confirm that the manuscript includes all relevant data to replicate the results of the study.

I confirm that all relevant data required to replicate the results of the study are included in the manuscript. Specifically, the manuscript provides all necessary data such as the values behind the means, standard deviations, and other measures, as well as the data used to construct the graphs and any points extracted from images for analysis.

If you require any additional information or further clarification on the data, please do not hesitate to let me know.

Q_5_: ORCID iD Requirement

A_5_: I have obtained an ORCID iD and have successfully validated it in Editorial Manager. Please let me know if there are any further steps I need to take regarding this.

**Ⅱ. Revisions Regarding Reviewer comments**

Thank you for the time and effort you dedicated to reviewing my manuscript. Your valuable suggestions have provided me with new insights on how to improve the article and guide my future research. In my response, I will address each of your comments individually, and you can find my revisions and explanatory notes on the structural corrections in the resubmitted document.

**The core issue of the research is** the real-time and precise regulation of airflow volume in the ventilation network. **The main focus is** that the traditional airflow regulation model, which considers natural ventilation pressure and ventilation resistance as constant values, making it unable to determine the optimal solution set for airflow regulation. Consequently, precise airflow regulation cannot be achieved. **The key to precise ventilation control is** solving the fluctuating airflow state properties in time series based on the heat and airflow coupling characteristics, deriving the fluctuating natural ventilation pressure and ventilation resistance, and applying a nonlinear correction to the traditional airflow regulation model to obtain the optimal solution set.

**The main research focus of Chapter 2** is to propose a density-pressure-temperature coupling equation that describes the variation patterns of airflow state properties, forming a transient heat and airflow flow characteristics model in time series. By incorporating this characteristics model as a correction variable, further develops a fluctuating natural ventilation pressure and ventilation resistance model in time series. Finally, based on the heat and airflow coupling characteristics of airflow, a nonlinear airflow regulation model is constructed to optimize airflow regulation.

**Chapter 3 focuses on** verifying the reliability of the proposed transient heat and airflow flow characteristics model and the heat and airflow coupling characteristics of airflow. This is achieved through CFD simulation, solving a full-scale unsteady-state heat transfer model based on the complex environmental and gradient flow fields of an actual mine.

**Chapter 4 applies** the research findings to a real-world case study, using the Gucheng Coal Mine as the research subject. Field-surveyed complex environmental and gradient flow fields are used as constraints and are integrated into the proposed transient heat and airflow flow characteristics model and fluctuating natural ventilation pressure and ventilation resistance model. This allows for the calculation of airflow state properties values and fluctuating values of natural ventilation pressure and ventilation resistance. The computed values are then input into the airflow regulation model to determine the optimal ventilation regulation scheme for the Gucheng Coal Mine in time series. This enables real-time and precise control of airflow volume in the ventilation network, thereby validating the core issue of the research.

**ⅡA). Reviewer #1**

Q_1_: In Section 2.2, a numerical model of unsteady heat transfer for airflow in the Gucheng Coal Mine is presented. It is recommended to incorporate field measurement data or experimental data to validate the accuracy of the simulation model.

A_1_: I conducted field measurements and calculations of the length, area, perimeter, height difference, roadways temperature, and frictional resistance of underground roadways before performing simulations. I also monitored and calculated the atmospheric pressure, atmospheric temperature, and the air pressure and air volume of the fans. Based on these data, I constructed the simulation model, which can be considered accurate within an acceptable error range. However, I did not mention the model parameter values in the article. In response to your suggestion, I have compiled the data and added a basic parameter table in Section 3.2 of the article (as shown below).

Table 1. Initial physical conditions of model

| Equivalent branch | Function | Length  *L* /m | Altitude difference  *Z* /m | Equivalent area  *S* /m^2^ | Equivalent hydraulic diameter  *d*_h_ /m | Thermal conductivity of wall layer  *λ*_k_ /(W·m^-1^·K^-1^) | Surface roughness  *η /m* |
| --- | --- | --- | --- | --- | --- | --- | --- |
| *e*_1_ | the central inlet shaft | 500 | 500 | 34.11 | 6.59 | 2.75 | 7.02×10^-1^ |
| *e*_2_ | the inlet airpath for the eastern and western areas of the mine from the central inlet shaft | 3000 | 0 | 32.37 | 6.42 | 0.31 | 2.59×10^-3^ |
| *e*_3_ | the Taoyuan inlet shaft | 500 | 500 | 29.51 | 6.13 | 2.75 | 9.40×10^-1^ |
| *e*_4_ | the Taoyuan inlet shaft to the southern inlet airpath | 5000 | 0 | 31.77 | 6.36 | 0.31 | 1.33×10^-4^ |
| *e*_5_ | the central inlet shaft to the southern inlet airpath | 3910 | 0 | 28.18 | 5.99 | 0.31 | 6.40×10^-^^4^ |
| *e*_6_ | the outlet airpath from the eastern and western areas to the central outlet shaft | 5000 | 0 | 29.42 | 4.81 | 0.31 | 1.07×10^-3^ |
| *e*_7_ | the southern outlet airpath | 3000 | 0 | 33.08 | 5.10 | 0.31 | 1.66×10^-3^ |
| *e*_8_ | the central outlet shaft | 500 | -500 | 64 | 8 | 2.75 | 1.03 |
| *e*_9_ | the Taoyuan outlet shaft | 500 | -500 | 64 | 8 | 2.75 | 9.44×10^-1^ |

During the actual survey, it was found that most of the main roadways underground were coal roadways. Therefore, when modeling, the wall boundary material of the shaft was set as siltstone, and the boundary material of the other roadways was set as lean coal.

The parameter differences between coal roadways and rock roadways are reflected in the heat transfer parameters and wall layer temperature of the roadway. The most direct heat transfer parameter affecting the convective heat transfer between the airflow and the roadway is the thermal conductivity of the wall layer, *λ*_k_ , and the original temperature of the wall layer (since the unstable heat transfer coefficient needs to be calculated through *λ*_k_ . The unstable heat transfer coefficient is the main calculation item for calculating the heat exchange magnitude between the airflow and the wall layer. Other parameters such as specific heat and density are automatically coupled and set when the material is modeled, and do not need to be assigned separately).

I have listed the thermal conductivity parameters, *λ*_k_, of the wall layer in the table. It can be seen from the table that there is a significant difference in the thermal conductivity between the coal layer and the rock layer. In Section 3.3: Boundary Condition Settings, I have drawn the distribution of the underground temperature field. From the figure, it can be seen that under the influence of geothermal heat flow, the temperature setting of the coal roadway is higher than that of the rock roadway.

Fig.5 Temperature distribution in mine roadways under the underground environmental field

Q_2_: In Section 2.3, The proposed airflow control model, which is based on the heat-flow coupling characteristic of airflow, should be compared with currently commonly used control models. It is suggested that the proposed model be compared with commonly used control models to clearly explain the advantages and disadvantages of the new model.

A_2_: In traditional ventilation system analysis, airflow is treated as an incompressible turbulent fluid, and the airflow in roadways is studied as independent entities. For each roadway, a single density value is measured and used as a constant density, with the corresponding natural ventilation pressure and ventilation resistance also being constant values. At this stage, the ventilation system operation can be considered a closed system with an isochoric process. Based on this, the fundamental construction principle of the commonly used airflow regulation models is to treat ventilation resistance and natural ventilation pressure as constant environmental parameters, and to use air volume and adjustment resistance as optimization decision variables. The conservation laws of air volume and air pressure serve as nonlinear equality constraints to complete the nonlinear airflow regulation model setup. In other words, airflow regulation focuses on the dynamic airflow process in the mine, treating the airflow as an isochoric process characterized by constant density.

However, under the influence of unsteady environmental and flow fields, the airflow essentially undergoes variable heat flow under the coupling of aerodynamic and thermal effects, with the state properties of the airflow constantly in a dynamic equilibrium state (referenced from Shengqiang Yang's "Research on thermal and dynamic changes of airflow and thermal resistance in high-temperature and high-humidity mines"). The natural ventilation pressure and ventilation resistance calculated based on the constant state property values of the isochoric process are constant values in time series. Constructing airflow regulation models with constant natural ventilation pressure and ventilation resistance as environmental parameters cannot determine the optimal set of airflow regulation solutions to achieve precise control of ventilation network air volume. Therefore, the core of constructing the airflow regulation model is to establish a equation for the dynamic changes in the airflow state properties (temperature, density, and pressure) in time series.

This paper constructs a transient heat and airflow flow characteristics model and a fluctuating natural ventilation pressure and ventilation resistance model by analyzing the dynamic changes in the state properties of mine airflow in time series, aiming to solve the fluctuating values of natural ventilation pressure and ventilation resistance in time series. By treating fluctuating natural ventilation pressure and ventilation resistance as environmental variables, adjustment resistance as decision variables, and minimizing system power consumption as the objective function, an airflow regulation model based on the heat and airflow coupling characteristics of airflow is constructed.

Compared to traditional airflow regulation model, the airflow regulation model based on the heat and airflow coupling characteristics, which integrates the transient heat and airflow flow characteristics model and the fluctuating natural ventilation pressure and ventilation resistance model, has the following advantages:

1.Consideration of Real-Time Environmental Dynamics: Traditional models typically assume constant state property values for airflow, which is not suitable for the real-time changes in the unsteady environmental and flow fields in actual production mines. In contrast, the model proposed in this paper establishes a transient heat and airflow flow characteristics model based on the dynamic changes in the airflow state properties (such as temperature, density, and pressure) in time series, capturing the changes in airflow state over time and avoiding the limitations of static environmental assumptions in traditional models.

2.Precision Control: Traditional models rely on constant natural ventilation pressure and ventilation resistance for airflow regulation, which cannot adjust according to actual environmental changes, leading to poor accuracy in air volume regulation. The model proposed in this paper dynamically calculates the fluctuating values of natural ventilation pressure and ventilation resistance, using them as environmental variables, combined with adjustable resistance as decision variables, to achieve precise optimization of airflow regulation, allowing real-time adjustment of ventilation network air volume according to actual working conditions.

Based on your suggestion, I have analyzed and summarized the main directions and achievements of existing airflow regulation technology research in Section 1: Introduction, and proposed that "these studies focus on the dynamic airflow process in mines, treating the airflow as an isochoric process characterized by constant density, and constructing and solving airflow regulation models with constant natural ventilation pressure and ventilation resistance as environmental parameters, based on the dynamic properties of airflow." Additionally, I have added a direction for improving existing airflow regulation models in the introduction to lead into the corrected airflow regulation model based on the heat and airflow coupling characteristics. "Natural ventilation pressure and ventilation resistance calculated based on constant state property values of the isochoric process or constant state property values at specific moments are constant values in time series. Constructing airflow regulation models with constant natural ventilation pressure and ventilation resistance as environmental parameters cannot determine the optimal set of airflow regulation solutions to achieve precise control of ventilation network air volume. Therefore, the key to precise airflow control is to solve for the fluctuating values of airflow state properties in time series based on the heat and airflow coupling characteristics, calculate the fluctuating natural ventilation pressure and ventilation resistance, and use this to nonlinearly correct the airflow regulation model and solve for the optimal set of model solutions.Based on this, the main implementation method of the research is proposed: by analyzing the dynamic changes in the state properties of mine airflow in time series, constructing a transient heat and airflow flow characteristics model and a fluctuating natural ventilation pressure and ventilation resistance model, to solve for the fluctuating values of natural ventilation pressure and ventilation resistance in time series. Treating the fluctuating natural ventilation pressure and ventilation resistance as environmental variables, adjustable resistance as decision variables, and minimizing system power consumption as the objective function, a corrected airflow regulation model based on the heat and airflow coupling characteristics of airflow is constructed. By solving this model, the optimal set of airflow regulation solutions is obtained, ultimately achieving precise control of ventilation network air volume, providing theoretical basis and technical support for improving the reliability of mine ventilation systems."

Additionally, in Section 2.3: Corrected airflow regulation model in time series, after constructing the corrected airflow regulation model based on the heat and airflow coupling characteristics of airflow, I added a paragraph to explain the advantages of the proposed model. "The airflow regulation model based on the heat and airflow coupling characteristics, which integrates the transient airflow flow characteristics model and the fluctuating natural ventilation pressure and ventilation resistance model, can accurately describe the changes in airflow state over time, respond to environmental changes in real-time, and dynamically adjust the airflow state accordingly. This optimizes air volume distribution and effectively meets the adaptive regulation requirements of mine ventilation systems in complex dynamic environments."

Q_3_: In Section 3.1, there are errors in the notation of Equations 14 and 15; please review and correct them. In Section 3.2, the ventilation resistance calculation for the central ventilation network and Taoyuan ventilation network in Scheme 2.1 are incorrectly written; please check and amend accordingly.

A_3_: The original equations (14) and (15) in Section 3.1 are the momentum and energy equations for turbulence. Since the underground airflow is turbulent, the Reynolds averaging method was used to decompose the flow velocity into mean velocity and irregular fluctuating velocity. Accordingly, the relevant terms in the momentum and energy conservation equations were modified, and turbulence was considered as an additional Reynolds stress to account for its impact on airflow motion and energy transfer.Regarding the issue you raised about errors in the equations, after careful examination, I found that additional Reynolds stress, pressure, and flow velocity were not set as vectors, and the multi-directional gradient symbol was incorrectly used. After thorough inspection, these symbol errors have been corrected, and the relevant equations have been updated. Additionally, due to significant modifications in the numerical simulation section of the article, the original equations (14) and (15) have been merged into the thermal flow model set in Section 3.1.

$$\left\{\begin{matrix} \rho SC_{\text{p}}\partial T/\partial t+\rho SC_{\text{p}}\mathbf{ue}\cdot\nabla T=\nabla\cdot\left(S\lambda_{\text{k}}\nabla T\right)+\left(1/2\right)f_{\text{D}}\left(\rho S/d_{\text{h}}\right)\left|\mathbf{u}\right|\mathbf{u}^{2}+A+\left(hD\right)_{\text{eff}}\left(T_{\text{ext}}-T\right) \\ \rho\partial\mathbf{u}/\partial t=-\nabla p\cdot\mathbf{e}-\left(1/2\right)f_{\text{D}}\left(\rho/d_{\text{h}}\right)\left|\mathbf{u}\right|\mathbf{u}+\mathbf{F}\cdot\mathbf{e} \\ \partial S\rho/\partial t+\nabla\left(S\rho\mathbf{ue}\right)=0 \\ \nabla\left(\rho\bar{\mathbf{u}}\bar{\mathbf{u}}\right)=\mathbf{F}-\nabla\left(\bar{P}\right)-\partial\left(\rho\bar{\mathbf{u}}\right)/\partial t+\nabla\left(\mathbf{\tau}+\mathbf{\tau}'\right) \\ \partial\left(\rho\varepsilon\right)/\partial t+\nabla\left(\rho\varepsilon\mathbf{u}\right)=\nabla\left(\mu\left(\nabla\mathbf{u}+(\nabla\mathbf{u})^{T}\right)-2/3\mu\nabla\mathbf{u}\right)+\varepsilon_{\text{k}}-\varepsilon_{\text{e}}+\varepsilon_{\text{p}} \end{matrix}\right.$$

Regarding the issue of errors in the ventilation resistance calculations for Central Ventilation Network and Taoyuan Ventilation Network in Scheme 2.1 of Section 3.2, the main focus of my previous paper was based on the variable flow process of airflow. By coupling density and pressure instead of using time as a variable, a coupling equation *ρ*=*k*(*f*(*P*, *ρ*) was formed to describe the variation of density during the airflow process. The calculation equation for natural ventilation pressure was corrected based on *ρ*=*k*(*f*(*P*, *ρ*). By solving the corrected natural ventilation pressure within the domains [*ρ*_min_, *ρ*_max_] and [*P*_min_, *P*_max_] for extreme values, these were used as intercept update factors to update the data for the fan performance curve equation. The ventilation network calculation was then redone, and by comparing the calculated data with the required airflow volume underground, it was found that when supplying air according to the updated conditions, the airflow in some branches did not meet the demand, thus requiring airflow regulation.

However, during the modification process, I found that variable density not only causes changes in natural ventilation pressure but also leads to dynamic changes in ventilation resistance by affecting the R value. Additionally, using density and pressure coupling instead of time as a variable can cause some data points to overlap in multiple dimensions, potentially leading to data distortion. Therefore, I reintroduced the time variable to construct a transient heat and airflow flow characteristics model *ρ*=*f*_3_(*n*, *P*, *T*, *t)* describing the dynamic changes in the state property values of airflow in time series. Based on this, I proposed a fluctuating natural ventilation pressure and ventilation resistance model to calculate the fluctuating values of natural ventilation pressure and ventilation resistance in time series.

At this point, the focus of the research shifted to real-time airflow regulation, rather than matching the updated operating points based on the extreme values of natural ventilation pressure with the airflow volume in underground branches. Therefore, the revised paper no longer involves the calculation of equivalent ventilation resistance for the ventilation network.

Q_4_: Figures 8 and 10 represent the fan characteristic curves. The working conditions and blade angles should be clearly labeled in the figures to help readers observe the fan's status before and after adjustment.

A_4_: The original Figures 8 and 10 are fan performance curve diagrams, corresponding to the operating points of the central fan and Taoyuan fan at Gucheng Coal Mine, as well as the fan performance characteristic curves at specific blade angles. Based on your suggestion, it is indeed necessary to clearly label the operating points and blade angles of different fans in these figures, so that readers can better observe the comparison of fan operating conditions before and after using the natural ventilation pressure extremes as intercept update factors.

However, during the revision process of this paper, the focus of the research has changed. The original study focused on the relationship between airflow regulation based on fan performance curves and fan performance. The current research direction has shifted to using the airflow regulation model based on the heat and airflow coupling characteristics for real-time optimal airflow regulation decision-making. To adapt to the new research focus, relevant parts of the paper have been adjusted. We have shifted the research focus from fan performance curves to the real-time dynamic adjustment of airflow state and optimization of air volume distribution. Therefore, the original Figures 8 and 10 are no longer applicable to the current research content.

Under the new research framework, we no longer focus on discussing the direct impact of fan performance curves and blade angles on airflow regulation. Instead, we aim to achieve real-time, precise airflow regulation by constructing a transient heat and airflow flow characteristics model and a fluctuating natural ventilation pressure and ventilation resistance model. Considering the deviation of Figures 8 and 10 from the current research content of this paper, we have decided to delete these two figures to avoid causing confusion for the readers.

Q_5_: The units throughout the paper should be consistent, and variables in the equations should be defined. The figures and textual descriptions should be as clear and understandable as possible. Some figures are unclear; it is advised that the author revise them to improve clarity.

A_5_: Regarding the issues of unit consistency and variable annotation in the paper, I have thoroughly reviewed and revised the entire text. All units have been standardized, and variables are clearly defined within the equations to avoid any potential ambiguity.

Concerning the textual expression issues, I have read through the entire paper and identified certain formatting errors, grammatical mistakes, and awkward sentences. I have rectified the coherence of the sentences, split complex and obscure long sentences, and rewritten them. All modifications have been marked in the submitted file "Revised Manuscript with Track Changes."

For the issues with non-standard figures and tables, I have conducted a detailed review and made corrections to the relevant figures and table legends in the paper. All images and tables have been reformatted as required, and any unclear descriptions have been rectified with detailed annotations. The specific changes are also marked in the uploaded file "Revised Manuscript with Track Changes." Please review them.

Q_6_: In the conclusion section, while the main findings and contributions of the study should be clearly stated, the practical significance of these findings for the design, operation, and decision-making of intelligent ventilation systems should also be highlighted. The paper currently lacks a detailed discussion on the practical implications of the work, which should be addressed in the conclusion.

A_6_: During the revision process, it was found that the original conclusion did not comprehensively summarize the research content and failed to describe the practical implications of the study. Therefore, the conclusion section has been extensively revised. The revised conclusion is as follows:

Content Summary:

1. To precise airflow control, three models are sequentially proposed: a transient heat and airflow flow characteristics model of airflow to quantitatively describe airflow state variations in time series; a fluctuating natural ventilation pressure and ventilation resistance model to quantitatively characterize their fluctuation patterns and impacts on ventilation systems; and an airflow regulation model to quantitatively determine optimal airflow regulation solution in time series.

Research Significance:

2. The constructed time-series-based airflow regulation model incorporating heatand airflow coupling characteristic can accurately capture dynamic changes in airflow state properties and ventilation parameters. By real-time quantification of fluctuations in natural ventilation pressure and ventilation resistance caused by dynamic mine environmental characteristics, the model enables optimal real-time airflow regulation decisions at different time steps, achieving precise real-time ventilation network airflow regulation. This real-time precise airflow regulation reduces unnecessary air volume waste, thereby decreasing ventilation system power consumption and enhancing overall system economic efficiency.

Application Extension:

3. Real-time precise airflow regulation dynamically responds to mine environmental changes, effectively improving the adaptive control accuracy and response speed of ventilation systems while optimizing control energy efficiency of the ventilation network. This approach provides crucial data support for the automation and intelligent development of mine intelligent ventilation systems, establishing a technical foundation for intelligent mine ventilation through dynamic regulation mechanisms and time-series-based optimization methods.

**ⅡB). Reviewer #2**

Q_1_: In Section 3.1, the authors perform transient solving of the non-steady-state heat transfer model for the coal mine. How were the continuous data for temperature, pressure, and time in Table 2 derived?

A_1_: Computational Principle: The solution to the airflow process in unsteady environmental fields and gradient flow fields is transformed into the analysis of the internal energy conversion process of compressible airflow exchanging heat with the solid boundaries of the model. Based on Fourier's law and Newton's law of cooling, the analysis of the internal energy changes of airflow due to heat exchange is converted into the numerical solution of airflow temperature, pressure, and density.

By simultaneously solving the momentum, energy, and mass conservation equations, we construct the heat transfer balance equation, the non-isothermal flow equation, and the body force equation, forming a mathematical model for airflow heat flow. Solving this model yields the transient temperature field *T*(*x*, *y*, *z*, *t*), pressure field *P*(*x*, *y*, *z*, *t*), and density field *ρ*(*x*, *y*, *z*, *t*). The heat flow model is equationted based on the Reynolds-averaged method (as shown in Equation below)：

$$\left\{\begin{matrix} \rho SC_{\text{p}}\partial T/\partial t+\rho SC_{\text{p}}\mathbf{ue}\cdot\nabla T=\nabla\cdot\left(S\lambda_{\text{k}}\nabla T\right)+\left(1/2\right)f_{\text{D}}\left(\rho S/d_{\text{h}}\right)\left|\mathbf{u}\right|\mathbf{u}^{2}+A+\left(hD\right)_{\text{eff}}\left(T_{\text{ext}}-T\right) \\ \rho\partial\mathbf{u}/\partial t=-\nabla p\cdot\mathbf{e}-\left(1/2\right)f_{\text{D}}\left(\rho/d_{\text{h}}\right)\left|\mathbf{u}\right|\mathbf{u}+\mathbf{F}\cdot\mathbf{e} \\ \partial S\rho/\partial t+\nabla\left(S\rho\mathbf{ue}\right)=0 \\ \nabla\left(\rho\bar{\mathbf{u}}\bar{\mathbf{u}}\right)=\mathbf{F}-\nabla\left(\bar{P}\right)-\partial\left(\rho\bar{\mathbf{u}}\right)/\partial t+\nabla\left(\mathbf{\tau}+\mathbf{\tau}'\right) \\ \partial\left(\rho\varepsilon\right)/\partial t+\nabla\left(\rho\varepsilon\mathbf{u}\right)=\nabla\left(\mu\left(\nabla\mathbf{u}+(\nabla\mathbf{u})^{T}\right)-2/3\mu\nabla\mathbf{u}\right)+\varepsilon_{\text{k}}-\varepsilon_{\text{e}}+\varepsilon_{\text{p}} \end{matrix}\right.$$

Here, *C*_p_ is specific heat at constant pressure, J/(kg∙K); ***e*** is Tangential vector; ***u*** is Velocity vector, m/s; *λ*_k_ is thermal conductivity of the surrounding rock, W/(m·K); *f*_D_ is Darcy frictional factor; *d*_h_ is hydraulic diameter of the model pipe, m; *A* is heat source, W∙m; *h* is heat transfer coefficient, W/(m^2^·K); *D* is wetted perimeter of the pipe, m; *T*_ext_ is temperature on the outer side of the pipe wall, K; ***F*** is body force, N; *μ* is dynamic viscosity, Pa∙s; ***τ*** is surface stress, N; $\bar{\mathbf{u}}$is mean velocity, m/s; $\mathbf{\tau}'$is additional Reynolds stress caused by airflow pulsation, N; *ε* is turbulent dissipation rate, m^2^∙s^3^; *ε*_k_,is dissipation of turbulent kinetic energy; *ε*_e_ is momentum dissipation caused by pressure gradient; *ε*_p_ is additional momentum dissipation caused by pulsating velocity.

In an actual mine, when airflow moves through the roadways, friction with the wall surfaces generates resistance. Therefore, it is necessary to account for the pressure loss due to airflow in a rough pipe model. Using the Darcy–Weisbach equation, by setting the pressure drop value equal to the actual ventilation frictional resistance value, the equation for the pressure loss along the length of airflow in a rough pipe model is established (Equation 2). The relationship between the Darcy frictional factor *f*_D_ and the Reynolds number is used to calculate the wall roughness *η* of the model (calculation equation shown in Equation 3).

$\Delta p=f_{\text{D}}\left(L/d_{\text{h}}\right)\left(\rho\left(\mathbf{u}\cdot\mathbf{e}\right)^{2}/2\right)=\left(\lambda\rho LU/8S^{3}\right)Q^{2}=\lambda\left(L/d_{\text{h}}\right)\left(\rho\left(\mathbf{u}\cdot\mathbf{e}\right)^{2}/2\right)$(2)

$f_{\text{D}}=8\left(\left(8\mu/\rho\mathbf{u}d_{\text{h}}\right)^{12}+\left(\left(-2.46\ln\left(\left(7\mu/\rho\mathbf{u}d_{\text{h}}\right)^{0.9}+0.27\left(\eta/d_{\text{h}}\right)\right)\right)^{16}+\left(37530\mu/\rho\mathbf{u}d_{\text{h}}\right)^{16}\right)^{-1.5}\right)^{1/12}$(3)

Here,∆*p* is pressure drop, Pa; *η* is surface roughness,m.

Simulation Calculation: To solve the above problem in the CFD simulation software, add a transient study step in the "Study" module. Configure the time-dependent transient solver, set the start time to 0h, the end time to 23h, and the output time step to 1h (the time step setting has been independently verified using a stepwise test method unrelated to the Courant number). Set global scaling with an absolute tolerance factor of 0.1, use the backward differentiation equation (BDF) for time stepping, with a maximum BDF order of 5 and a tolerance of 0.001. Use the MUMPS model and constant Newton method for fully coupled linear and nonlinear solutions, ending the iterative computation when the convergence tolerance of 0.001 is reached. Click "Compute" in the study module to run the transient solver.

Image Export: In the "Results" module, add a temperature plot group, select surface plot, choose the study solution as the dataset, and set the expression to temperature *T.* Select the required roadway/global roadway, plot the temperature variation over time, and right-click to export the image.

Data Export: In the "Definitions" module, add probes (to record the simulation data of selected region physical quantities as a function of time). Set the temperature, pressure, and density of each roadway as probe monitoring variables, configure the probes to average in the form of 4th-order quadrature points. After setting up the probes, run the simulation to obtain the physical quantity data at each time step probe location.

In summary, I represent the heat flow of the airflow using a mathematical model, construct space-discretized finite element equations over time steps to solve for *T*(*x*, *y*, *z*, *t*), pressure *P*(*x*, *y*, *z*, *t*), and density *ρ*(*x*, *y*, *z*, *t*). Using CFD simulation technology, I solve for the state values at each time step, integrate them to form continuous time series, and obtain continuous data of temperature, pressure, and time under continuous spatiotemporal characteristics.

Q_2_: The grid has a significant impact on the results. In the grid independence verification for the heat transfer model, the grid evaluation parameters used in Figure 6 continue to rise as the number of grids increases. An additional analysis of the selected grid division scheme should be included. It is also suggested to adjust the content of Figure 6 to ensure that the curve remains complete.

A_2_: When dividing the mesh, I first used the sweeping operation to perform structured mesh division for the regular regions. Then, for the remaining parts, I performed free meshing. The remaining meshing adopted adaptive sweeping mesh. Additionally, due to the presence of temperature boundary layers and flow boundary layers at the model walls, I added prism boundary layer meshes to the airflow flow to reduce errors and accurately display the gradient changes of the physical field.

For mesh independence verification, I mainly used parametric scanning. I provided a parameter list of acceptable maximum element sizes [50, 40, 30, 20, 10]. When the size parameter varied within [20, 10], the changes in mesh evaluation parameters tended to stabilize. I further modified the parameter variation intervals to [1, 0.5, 0.3, 0.2, 0.1], resulting in different mesh division outcomes. When the maximum element size was 18.7, the mesh count was 586,100, and the changes in mesh evaluation parameters had reached the convergence standard. Therefore, I chose this mesh size. However, upon further inspection, I realized that the mesh should be refined further because the rate of change between 586,100 and 613,800 was too large to determine if convergence was achieved. The mesh data for Figure 6 is shown in Table 1 below.

Table 1. Mesh independence verification

| mesh density | mesh evaluation parameters/K | rate |
| --- | --- | --- |
| 18.43万 | 302.08 | / |
| 35.23万 | 301.96 | 0.04% |
| 52.81万 | 301.23 | 0.24% |
| 58.61万 | 300.87 | 0.12% |
| 61.38万 | 300.85 | 0.007% |

After receiving the revision comments, I found that most of the modifications were concentrated in the numerical simulation module. Therefore, I comprehensively revised and redid the simulation. I re-meshed the model based on the original solid model, without changing the meshing scheme and independence verification method. The initial mesh count was increased from 586,100 to 790,000.

To enhance mesh uniformity and improve simulation accuracy, I refined the parameter list for the mesh gradation rate to [1.05, 1.04, 1.03, 1.02, 1.01], still using the temperature of e1 as the evaluation parameter, and raised the convergence standard to E-5. The results of the mesh independence verification are shown in Table 2 below. Combining Table 1 and Table 2, it can be concluded that when the mesh count is 790,000, the temperature change rate meets the convergence standard. Additionally, changing the mesh gradation rate values does not affect the simulation results, indicating that the mesh is independent of the simulation results at this point.

Table 2. Temperature distribution of e1 in time series after changing the mesh gradient rate

| Times | 1.05 | 1.04 | 1.03 | 1.02 | 1.01 |
| --- | --- | --- | --- | --- | --- |
| 0 | 297.34 | 297.34 | 297.34 | 297.34 | 297.34 |
| 1 | 293.19 | 293.19 | 293.19 | 293.19 | 293.19 |
| 2 | 292.56 | 292.56 | 292.56 | 292.56 | 292.56 |
| 3 | 292.02 | 292.02 | 292.02 | 292.02 | 292.02 |
| 4 | 291.79 | 291.79 | 291.79 | 291.79 | 291.79 |
| 5 | 291.57 | 291.57 | 291.57 | 291.57 | 291.57 |
| 6 | 291.74 | 291.74 | 291.74 | 291.74 | 291.74 |
| 7 | 292.01 | 292.01 | 292.01 | 292.01 | 292.01 |
| 8 | 292.3 | 292.3 | 292.3 | 292.3 | 292.3 |
| 9 | 292.62 | 292.62 | 292.62 | 292.62 | 292.62 |
| 10 | 292.9 | 292.9 | 292.9 | 292.9 | 292.9 |
| 11 | 293.01 | 293.01 | 293.01 | 293.01 | 293.01 |
| 12 | 293.01 | 293.01 | 293.01 | 293.01 | 293.01 |
| 13 | 294.47 | 294.47 | 294.47 | 294.47 | 294.47 |
| 14 | 296.07 | 296.07 | 296.07 | 296.07 | 296.07 |
| 15 | 296.6 | 296.6 | 296.6 | 296.6 | 296.6 |
| 16 | 296.45 | 296.45 | 296.45 | 296.45 | 296.45 |
| 17 | 296.09 | 296.09 | 296.09 | 296.09 | 296.09 |
| 18 | 295.27 | 295.27 | 295.27 | 295.27 | 295.27 |
| 19 | 294.46 | 294.46 | 294.46 | 294.46 | 294.46 |
| 20 | 293.81 | 293.81 | 293.81 | 293.81 | 293.81 |
| 21 | 293.28 | 293.28 | 293.28 | 293.28 | 293.28 |
| 22 | 293.24 | 293.24 | 293.24 | 293.24 | 293.24 |
| 23 | 293.38 | 293.38 | 293.38 | 293.38 | 293.38 |

Fig 1. Temperature distribution of *e*1 under multiple grid gradient rates

Considering the length of the article, the mesh division module was directly removed. The mesh independence verification in the revision notes is provided for review purposes. If, after your review, you believe that this module is meaningful and should be included in the article, my alternative plan is to describe the mesh independence verification module directly in the text as follows:"Mesh independence verification was conducted by using parametric scanning to modify element size and mesh gradation rate. When the mesh division size was 790,000 domain elements and the mesh gradation rate was 1.03, both computational accuracy and speed were balanced."

Q_3_: How is the proposed airflow regulation model applied in conjunction with CFD numerical simulation software? Please provide more details.

A_3_: In traditional ventilation system analysis, airflow is typically considered as an incompressible turbulent fluid, and the airflow in each roadway is studied as an independent entity. For each roadway, a constant density value is measured, treating the ventilation system operation as an isochoric process in a closed system. On this basis, the basic construction principle of commonly used airflow regulation models is to treat ventilation resistance and natural ventilation pressure as constant environmental parameters, while airflow volume and adjustable resistance are treated as optimization decision variables. The conservation laws of airflow volume and loop ventilation pressure serve as nonlinear equality constraints, completing the setup of the nonlinear airflow regulation model. In other words, airflow regulation focuses on the dynamic flow process of mine airflow, viewing the airflow as an isochoric process characterized by constant density.

However, under the influence of unsteady environmental fields and flow fields, the actual airflow movement is a variable thermal flow under the coupled aerodynamic and thermal effects. The state properties of the airflow are in a constant dynamic equilibrium (referenced from Shengqiang Yang's "Research on Thermal and Dynamic Changes and Thermal Resistance of Airflow in High-Temperature and High-Humidity Mines"). The natural ventilation pressure and ventilation resistance calculated based on the constant state properties of the isochoric process are constant values in time series. An airflow regulation model built with constant natural ventilation pressure and ventilation resistance as environmental parameters cannot determine the optimal solution set for airflow regulation to achieve precise control of the ventilation network airflow. Therefore, the core of constructing an airflow regulation model is to establish the governing equations for the dynamically changing airflow state properties (temperature, density, and pressure) over time.

Based on the Goff-Gratch equation, the characteristics of the polytropic process, and the underground airflow density calculation equation derived from Dalton's law, I introduced a time variable and rewrote the time series as *ρ*(t)=(*n*(t), *P*(t), *T*(t)). Using *ρ*(t)=(*n*(t), *P*(t), *T*(t)) as the governing equation for the state properties of the airflow, I constructed a transient thermal flow characteristic model of the airflow. I then replaced the density *ρ* in the traditional natural ventilation pressure and ventilation resistance calculation equations with *ρ*(t), forming a fluctuating natural ventilation pressure and ventilation resistance model.

By treating the fluctuating natural ventilation pressure and ventilation resistance as environmental variables, the adjustable resistance as the decision variable, and minimizing system power consumption as the objective function, I constructed a modified airflow regulation model based on the thermal flow coupling characteristics of the airflow.

$$P\left(1/\rho\right)^{n}=\text{const}=P_{0}\left(1/\rho_{0}\right)^{n}\to n=\left(\ln P\left(t\right)-\ln P_{0}\right)/\left(\ln\rho\left(t\right)-\ln\rho_{0}\right)$$

$$\left\{\begin{matrix} \rho-f_{1}\left(P,T,t\right)=0 \\ n-f_{2}\left(P,\rho,t\right)=0 \end{matrix}\right.\to\rho=f_{3}\left(n,P,T,t\right)$$

However, the reliability of the equation *ρ*(t)=(*n*(t), *P*(t), *T*(t)) has not been verified, and it cannot be directly used to calculate underground parameters. Therefore, I took the Gucheng Coal Mine in actual production as the research object. By conducting field surveys and on-site data collection of the unsteady environmental field and gradient flow field in Gucheng Coal Mine, and using CFD numerical simulation software for full-scale solid modeling and real boundary condition settings, I completed the transient simulation calculations. Based on the calculated pressure, temperature, and density data sets, I verified the reliability of the equation *ρ*(t)=(*n*(t), *P*(t), *T*(t)) within the error range, as well as the thermal flow coupling characteristics of the airflow.

Once the equation was verified to be reliable, I used this equation, starting from the air inlet shaft as the initial node and using the atmospheric environment field as the initial node parameter, with the main heat sources being the heat dissipation from surrounding rocks and the self-compression heating of the air. I calculated the temperature, pressure, and density distribution of the flowing air at each underground node. Then, using the node integration method, I completed the calculation of the average temperature, pressure, and density in the roadways in time series, thus obtaining *ρ*(t) in time series. I replaced the density *ρ* in the natural ventilation pressure and ventilation resistance calculation equations with *ρ*(t), completing the calculation of the fluctuating values of natural ventilation pressure and ventilation resistance. Based on this, I used the simplex method to solve the airflow regulation model, achieving real-time optimal decision-making for airflow regulation (there is an error between the calculation results and the simulation results, which I believe is mainly due to humidity and gravitational acceleration. During the simulation process, to form an integrated airflow, it was not possible to set the airflow humidity for each roadway individually. I set the model airflow humidity to remain at 90% and the gravitational acceleration at 9.81. In the calculation process, I used the sectional method to set the airflow humidity for each type of roadway to reflect the humidity gradient).

In summary, the reliability of the constructed modified airflow regulation model based on the thermal flow coupling characteristics of the airflow will be verified using CFD simulation technology. After verifying the reliability of the modified model, real mine site data will be substituted into the modified model for actual calculations to achieve real-time precise airflow control.

Q_4_: The authors should consider the potential discrepancies between the simulation results and actual conditions, and evaluate the effectiveness of the simulation. More details should be provided, including the methods of data collection, as well as a thorough analysis of the experimental results, so that readers can assess the reliability and validity of the study.

A_4_: Before conducting the simulation, I performed field measurements and calculations of parameters such as the length, area, perimeter, height difference, wall temperature, and wall frictional ventilation resistance of the underground roadways. I also monitored and calculated the atmospheric pressure, atmospheric temperature, and the ventilation pressure and airflow of the fans above ground. Based on these data, I constructed the boundary conditions for the unsteady environmental field and gradient flow field and completed the construction of the full-scale simulation model for Gucheng Coal Mine.

The objective is to use CFD simulation technology to solve the full-scale physical unsteady heat transfer model based on the complex environmental field and gradient flow field of the actual mine, in order to verify the thermal flow coupling characteristics of the airflow and the reliability of the proposed transient thermal flow characteristic model of the airflow (as shown in the following equation). It is important to note that the simulation results will not be used as actual state property values for subsequent airflow regulation in the coal mine.

$$P\left(1/\rho\right)^{n}=\text{const}=P_{0}\left(1/\rho_{0}\right)^{n}\to n=\left(\ln P\left(t\right)-\ln P_{0}\right)/\left(\ln\rho\left(t\right)-\ln\rho_{0}\right)$$

$$\left\{\begin{matrix} \rho-f_{1}\left(P,T,t\right)=0 \\ n-f_{2}\left(P,\rho,t\right)=0 \end{matrix}\right.\to\rho=f_{3}\left(n,P,T,t\right)$$

However, during the simulation process, to ensure the integrated flow characteristics of the airflow, it was not possible to set the humidity for each roadway individually. Therefore, in my simulation, I ignored the heat and moisture transfer effects and directly set the airflow humidity to a constant value of 0.9 using environmental properties to simplify the model.

When verifying the reliability of the proposed airflow state property equation, I substituted the simulated temperature *T*(*x*, *y*, *z*, *t*) and pressure *P*(*x*, *y*, *z*, *t*) into the airflow state property equation to calculate the density (with the humidity in this equation being a constant value of 0.9). I then compared the accuracy and performed residual analysis with the simulated density *ρ*(*x*, *y*, *z*, *t*)(as shown in the figure below), thus concluding that the proposed equation is reliable.

During the simulation, the only inconsistency was the humidity setting compared to the actual mine. For the residual analysis, I did not use the actual mine humidity but instead used the set value of 0.9 to verify the equation's reliability to achieve the simulation's purpose.

Additionally, in response to your request for more details, I have rewritten the content of the numerical module, adjusted the structure, and added sections on the mathematical model explanation, physical model setting parameters, model solving methods, data collection methods, and simulation result analysis. This is to enable readers to evaluate the reliability and validity of the research. Due to the extensive changes in the article, it would be too cumbersome to list them all here. You can find my modifications in the submitted document "Revised Manuscript with Track Changes." Please review it!

Q_5_: The models and data results mentioned in the article should be presented clearly through figures and tables to aid reader comprehension. Each figure and table should include clear titles, legends, and annotations to facilitate interpretation.

A_5_: I have thoroughly revised and updated the figures and tables in the article to ensure they are clearer and easier to understand. I have redrawn several images, added necessary titles, legends, and annotations to the charts, ensuring that each chart clearly conveys its content and helps readers better understand the significance of the model and data results.

Regarding the issue of non-standard charts, I have conducted a detailed review and made necessary annotations and modifications to the relevant charts in the article. All images and tables have been reformatted and re-entered as required. For issues with unclear expressions, I have corrected them using detailed annotations. The specific modifications are also marked in the uploaded document "Revised Manuscript with Track Changes." Please review it.

Q_6_: The formatting of the article, such as line spacing and other elements, should be adjusted to ensure consistency throughout the manuscript. It is recommended to carefully review the entire document, including text, figures, and images, to ensure uniform formatting.

A_6_: In response to the formatting issues, I have revised the entire article to ensure consistent formatting throughout. All equations, images, and tables have been reformatted and re-entered uniformly, and any identified issues have been corrected.

Additionally, a thorough read-through of the article revealed some formatting errors, grammatical mistakes, and awkward sentences. I have revised the article for sentence coherence, splitting and rewriting convoluted long sentences to enhance readability. The revision process has been marked in the submitted document "Revised Manuscript with Track Changes." Please review it.

Q_7_: The conclusions do not fully summarize the research content and its innovations. It is suggested to refine and strengthen the conclusions to more effectively highlight the key contributions and novel aspects of the study.

A_7_: During the revision process, it was identified that the original conclusions inadequately summarized the research scope and failed to address practical applications. Substantial modifications were consequently implemented, with the revised conclusions structured as follows:

Content Synthesis:

(1) To precise airflow control, three models are sequentially proposed: a transient heat and airflow flow characteristics model of airflow to quantitatively describe airflow state variations in time series; a fluctuating natural ventilation pressure and ventilation resistance model to quantitatively characterize their fluctuation patterns and impacts on ventilation systems; and an airflow regulation model to quantitatively determine optimal airflow regulation solution in time series.

Research Significance:

(2) The constructed time-series-based airflow regulation model incorporating heatand airflow coupling characteristic can accurately capture dynamic changes in airflow state properties and ventilation parameters. By real-time quantification of fluctuations in natural ventilation pressure and ventilation resistance caused by dynamic mine environmental characteristics, the model enables optimal real-time airflow regulation decisions at different time steps, achieving precise real-time ventilation network airflow regulation. This real-time precise airflow regulation reduces unnecessary air volume waste, thereby decreasing ventilation system power consumption and enhancing overall system economic efficiency.

Application Extension:

(3)Real-time precise airflow regulation dynamically responds to mine environmental changes, effectively improving the adaptive control accuracy and response speed of ventilation systems while optimizing control energy efficiency of the ventilation network. This approach provides crucial data support for the automation and intelligent development of mine intelligent ventilation systems, establishing a technical foundation for intelligent mine ventilation through dynamic regulation mechanisms and time-series-based optimization methods.

**ⅡC). Reviewer #3**

After receiving your feedback, I began revising my paper. Previously, the main focus of my paper was based on the polytropic process of airflow, where density and pressure coupling replaced the time variable. This formed a coupling equation *ρ*=*k*(*f*(*P*, *ρ*) used to describe the variation of density during the airflow process. I modified the calculation equation for natural ventilation pressure based on *ρ*=*k*(*f*(*P*, *ρ*) and solved for the extrema of the modified natural ventilation pressure within the domains [*ρ*_min_, *ρ*_max_] and [*P*_min_, *P*_max_]. These extrema were used as intercept updating factors to update the fan performance curve equation. After recalculating the ventilation network and comparing the recalculated data with the required airflow volume data in the mine, I found that the airflow supply in some branches did not meet the demand when supplying air based on the updated conditions, thus necessitating airflow regulation.

However, your comment, **"The authors should remember that the resistance of a single branch and the total resistance of the ventilation network are functions of the variable air density. Therefore, it is recommended that the standardized resistance of branches and the standardized fan characteristics be considered,"** made me realize that variable density not only causes changes in natural ventilation pressure but also leads to dynamic changes in ventilation resistance by affecting the value of *R*. Moreover, using density and pressure coupling to replace the time variable can cause some data points to overlap in multiple dimensions, potentially leading to data distortion. Therefore, I reintroduced the time variable and constructed a transient heat and airflow coupling characteristics model *ρ*=*f*_3_(*n*, *P*, *T*, *t*) to describe the dynamic changes in the airflow state properties over time. Based on this, I proposed a fluctuating natural ventilation pressure and ventilation resistance model to calculate the fluctuating natural ventilation pressure and ventilation resistance values over time. At this point, the research focus shifted to real-time airflow regulation using the fluctuating natural ventilation pressure and ventilation resistance values rather than matching the updated working condition based on natural ventilation pressure extrema with the underground branch airflow.

I am very grateful for your valuable feedback during the review process. Your suggestions made me re-examine my paper and prompted me to reflect on the rationality of the original research focus. Under your guidance, I realized the shortcomings of the previous research direction and successfully identified a new research focus. Your feedback has been crucial in improving the quality of my paper, and I sincerely appreciate your help and support. I hope to have the opportunity to receive your valuable feedback again in the future.

- **Core comments**

Q_1_: the problem of the influence of natural ventilation pressure (NVP) on the airflow in a mine ventilation network and the characteristics of the fan's operation has been known and described in the literature since at least the first half of the last century.

A_1_: I have read the articles and books you recommended. Indeed, the impact of natural ventilation pressure on mine airflow and fan operating characteristics has been extensively studied by many experts, and it has been thoroughly discussed in numerous publications. However, I found that most existing research focuses on the quantitative analysis of the impact of natural ventilation pressure on airflow at specific moments. For example, in traditional mine main fan parameter planning activities, natural ventilation pressure is usually simplified and treated as a static linear equation with seasonal trends (as shown in this figure).

However, when airflow with heat and airflow coupling characteristics flows in the mine ventilation system, the complex and dynamically changing mine environment will cause the state properties of the airflow (temperature, pressure, and density) to change dynamically over time. This, in turn, leads to continuous fluctuations in natural ventilation pressure and ventilation resistance. In other words, the dynamically changing state properties of the airflow will cause natural ventilation pressure to no longer follow a linear pattern but instead form a transient fluctuation sequence.

At the same time, I found that existing research on airflow regulation focuses on the flow process of mine airflow, treating the airflow flow as an isochoric process characterized by constant density. Natural ventilation pressure and ventilation resistance are considered constant environmental parameters when constructing and solving airflow regulation models, based on the constant properties of airflow.

Therefore, I have shifted my research focus to: quantifying the impact of the heat and flow of airflow on natural ventilation pressure and ventilation resistance over time and incorporating this into the airflow regulation model. By making real-time decisions to find the optimal solution set for airflow regulation, precise control of the airflow volume in the ventilation network can be achieved.

Q_2_: Authors are reminded that in the introduction, they should refer to articles describing the influence of the compressibility of air NVP in mine ventilation networks.

A_2_: Since I have adjusted my research focus to "quantifying the impact of the heat and flow of airflow on natural ventilation pressure and ventilation resistance over time and incorporating this into the airflow regulation model, achieving precise control of the airflow volume in the ventilation network through real-time decision-making for optimal airflow regulation solutions," I have updated and revised the existing researches in the introduction section based on your suggestions. I have summarized and analyzed the researches in two modules.

**Module 1-** summarizes the technical methods of existing mine airflow regulation, concluding that these studies focus on the flow process of mine airflow, treating the airflow flow as an isochoric process characterized by constant density. Natural ventilation pressure and ventilation resistance are considered constant environmental parameters when constructing and solving airflow regulation models, and airflow regulation is based on the constant properties of airflow.

**Module 2-** summarizes the existing research on the effects of unsteady heat flow on natural ventilation pressure and ventilation resistance in mine airflow, concluding that these studies focus on the quantitative analysis of the impact of heat and airflow at specific moments on natural ventilation pressure and ventilation resistance. However, they have not yet quantified the impact of heat and airflow on natural ventilation pressure and ventilation resistance over time and incorporated it into airflow regulation models.

Q_3_: the work analyzes the influence of NVP on the operation of the main ventilation fan. Therefore, the article's title should indicate that this is a different way of approaching this issue.

A_3_: Based on your feedback, I have revised the article title to better reflect the unique perspective and methodology of the research. The new title is: "Decision on optimal airflow regulation solution set based on heat and airflow coupling characteristics of mine airflow in time series". This title emphasizes the dynamic coupling effects between heat and airflow characteristics of mine airflow in time series and their impact on mine airflow regulation.

Unlike traditional research that focuses on the impact of static natural ventilation pressure on the mine ventilation system, this study focuses on quantitatively analyzing the impact of heat and airflow coupling characteristics on the airflow state properties. By analyzing the dynamic changes in the airflow state properties over time, the study deeply explores how these changes affect natural ventilation pressure and ventilation resistance. Based on this, a precise airflow regulation model is proposed. Therefore, the newly proposed title explains the decision optimization problem and the research focus, aiming to accurately convey the core content of the research and highlight its innovations and methods.

- **Other major comments**

Q_1_: To avoid any confusion, it is recommended that the term' heat and airflow coupling' be used to clearly indicate the relationship between heat and airflow, rather than being associated with heat flow only.

A_1_: I have revised the manuscript accordingly and have unified the term to “heat and airflow coupling” throughout the text. This change was made to clearly indicate the relationship between heat and airflow, rather than associating it with heat flow alone. This ensures clarity and consistency in the terminology used across the paper. I appreciate your attention to detail.

Q_2_: The authors should remember that the resistance of a single branch and the total resistance of the ventilation network are functions of the variable air density. Therefore, it is recommended that the standardized resistance of branches and the standardized fan characteristics be considered. This understanding is crucial as it significantly impacts the results of the analysis at the Gucheng Coal Mine.

A_2_: The point you raised about the impact of variable density on the ventilation resistance of a single roadway and the total resistance of the ventilation network is the main reason I changed my research focus. This made me realize that variable density not only causes changes in natural ventilation pressure but also leads to dynamic changes in the ventilation resistance of a single roadway over time by affecting frictional resistance.

Regarding the issue of standardization, I have standardized the ventilation resistance of a single roadway, and the specific steps are shown below. However, concerning the standardization of fan characteristics using the total resistance of the ventilation network, since my research focus has shifted from the fan characteristic curve to real-time dynamic regulation of airflow state and optimization of airflow distribution, I no longer focus on the matching of working condition based on natural ventilation pressure extrema with the airflow of underground branches. Therefore, I did not further standardize the fan characteristics.

Instead, I have added a section in the paper to quantify the negative feedback impact of fluctuating natural ventilation pressure and ventilation resistance on the ventilation system (Section 2.2, (3) Quantifying the impact of fluctuation corrections on the ventilation system). This part focuses on how to quantify the impact of fluctuations in natural ventilation pressure and ventilation resistance on the performance of the ventilation system, which aligns more closely with my current research direction.

Regarding the standardization of ventilation resistance in a single roadway:

Based on the law of turbulent frictional resistance, the calculation equation for ventilation resistance in mine roadways is given in equation (7). From equation (7), it can be seen that for a standardized mine roadway, *R_i_* is proportional to the friction coefficient *α* ,Since *λ* in a fully turbulent state is only related to the relative roughness of the wall surface, the *λ* value for a standardized mine roadway is constant, making *α* proportional only to the airflow density *ρ*. Thus, solving for the fluctuating ventilation resistance in mine roadways essentially involves using *ρ*(*t*) to determine the dynamically fluctuating *α.*

$h_{i-\text{r}}=R_{i}q_{i}^{2}=\left(\alpha_{i}L_{i}U_{i}/S_{i}^{3}\right)q_{i}^{2}=\left(\lambda_{i}\rho_{i}L_{i}U_{i}/8S_{i}^{3}\right)q_{i}^{2}$ (7)

Here, *h_i_*_-r_ is the ventilation resistance of roadway *i,* Pa; *R_i_* is the frictional resistance of roadway *i*, N∙s^2^/m^8^; *q_i_* is the airflow volume passing through roadway *i,* m^3^/s; *α_i_* is the friction coefficient of roadway *i,* kg∙m^3^; *L_i_* is the length of roadway *i,* m; *U_i_* is the perimeter of the cross-section of roadway *i*,m; *S_i_* is the cross-sectional area of roadway *i,*m^2^; *λ_i_* is the experimental proportional coefficient of roadway *i*; *ρ_i_* is the density of the airflow passing through roadway *i,* kg/m^3^.

There are three methods for calculating the friction coefficient α: ①Using the relationship between the experimental proportional coefficient *λ* and the airflow density *α*=*λρ*/8. However, the practical *λ* value in a fully turbulent state is difficult to measure and can only be approximated using empirical equations, which introduces an unknown error (precise values cannot be obtained). ②Calculating the roadway ventilation pressure using precise barometers and inclined manometers in various segments of the underground roadway, combined with monitoring airflow to complete data inversion of frictional resistance *R* and friction coefficient *α*. ③Using lookup tables to match the *α* value under standard conditions (*ρ*=1.2) based on the type of support. In actual production mines, method 2 is generally used, while method 3 is used for the design of new mines. Regardless of the method, the calculated *α* value is for a specific moment. Using the *α* value of a specific moment to represent the *α* value in entire time series will inevitably lead to errors in resistance *h*_r_, which need correction.

According to *α*=*λρ*/8, *α* is proportional to *ρ* (with *λ* as a constant for a given roadway type). Therefore, I use the transient heat and airflow flow characteristics model *ρ*=*f*_3_(*n*, *P*, *T*, *t*) in time series as a correction variable. By constructing the ratio of *ρ*(t) to the original specific moment *ρ*, corrections are made based on the specific moment *α* value, thereby correcting *h*_r_ and achieving the standardization of ventilation resistance for a single roadway. The values of *h*_r_、R、*α* and *ρ* at the specific moment are used as default for correction, with method 2 being used for solving. The specific moment *ρ* can be calculated using relative humidity and dry temperature detected by precise barometers.

Additionally, for reliability analysis, I have summarized the content of Section 2.2. By analyzing the proportional relationships between *h*_r_ - R, R- *α*, and *α* - *ρ*, I explained why parameter *α* was chosen for correction (α is the root variable directly affected by dynamic changes in ρ, completing parameter selection). I stated the three methods for calculating *α*, noting that method 1 is rarely used in actual mines due to its inherent error, while methods 2 and 3 both require correction. Method 2 uses the accurate values of *h*_r_、R、*α* and *ρ* at a specific moment as a reference to correct the values in time series. Method 3 involves correcting the theoretical values of *h*_r_、R、*α* and *ρ* under standard conditions (*ρ*=1.2) across all time states, demonstrating the universality of the correction method.

Q_3_: The particle swarm optimization algorithm for solving the ventilation network should be referred to the reference or described in more detail.

A_3_: In my previous research, I indeed used the Particle Swarm Optimization (PSO) algorithm to solve for the extremum of the corrected natural ventilation pressure within the domains of [*ρ*_min_, *ρ*_max_] and [*P*_min_, *P*_max_]. By using the extremum of the natural ventilation pressure as an intercept update factor, the performance curve equation of the fan was updated. This achieved the primary research objective: to quantitatively analyze the matching degree between the updated working condition based on the extremum of natural ventilation pressure and the demand airflow of underground branches, and to propose corresponding airflow regulation schemes.

However, in the current study, the research focus has shifted to the airflow regulation model based on the heat and airflow coupling characteristics of mine airflow. Therefore, the PSO algorithm is no longer the core technology for solving the current model. The new research method focuses on analyzing the interaction between dynamic environmental variables (fluctuating natural ventilation pressure and ventilation resistance) and decision variables (adjustment resistance) to construct and solve the airflow regulation model based on the heat and airflow coupling characteristics of mine airflow. Within this framework, we directly adopted methods such as the Simplex method to optimize system power consumption, ensuring a more efficient ventilation system design. Consequently, the PSO algorithm was not used in this study and is no longer the primary solving tool. Therefore, I did not describe the PSO algorithm in detail.

However, I understand that your suggestion essentially requires a detailed description of the research methods used to solve the model in the article. Therefore, in Section 2.4: Solving the Airflow Regulation Model, I have provided a detailed explanation of my research methods.

**2.4 Solution of the airflow regulation model**

From the perspective of mathematical logic, the core of solving the objective function of the constructed airflow regulation model, which is based on the heat and airflow coupling characteristics, lies in analyzing the interaction between the dynamic environmental variables and the decision variable. Based on the interaction mechanism between the variables, the objective function, min *N =* min *N*_1_+min *N*_2_, is divided into a multidimensional function *N*_1_ and a linear programming model function *N*_2_ using the fractional step method (as shown in Equation (16)). The decomposed *N*_1_ primarily focuses on analyzing the impact of fluctuating natural ventilation pressure and ventilation resistance on the power consumption of the ventilation system, reflecting the constraints imposed by dynamic underground environmental conditions on airflow. On the other hand, *N*_2_ focuses on the optimization process of adjustment resistance, highlighting the role of ventilation system design parameters in regulating power consumption. By decomposing the objective function, the independent optimization of environmental variables and decision variables is achieved, directly reducing the complexity of solving the model.

$\min N_{1}=\min\sum_{i=1}^{v} \left(\left(h_{i-\text{r}}\left(t\right)^{'}+h_{\text{N}}\left(t\right)^{'}\right)\left(q_{i}\left(t\right)^{'}\right)\right)$，$\min N_{2}=\min\sum_{i=1}^{v} \left(\Delta h_{i}\left(t\right)q_{i}\left(t\right)^{'}\right)$ (16)

The basic solution criterion for *N*_1_ is to calculate the instantaneous power consumption of the dynamically changing ventilation system at each time step, aiming to minimize the total system power consumption by optimizing the instantaneous consumption values. Specifically, solving *N*_1_ involves dynamic modeling of the airflow state in time series, ensuring that changes in natural ventilation pressure, ventilation resistance, and airflow at each time step comply with the law of conservation of mass and other physical constraints. Numerical methods are used to find the optimal power consumption of the system by optimizing the airflow distribution at each time step to achieve minimal power consumption.

The focus of solving *N*_2_ is on optimizing the impact of adjustment resistance on the power consumption of the ventilation system. By applying the simplex method to solve the linear programming function, multiple sets of *N*_2_ solutions, solution sets for different time steps are obtained, with each set representing the optimal adjustable resistance branch set {Δ*h_i_*} at the current time step. By gradually adjusting the resistance in real-time, the overall power consumption of the system is further reduced while meeting the airflow requirements of each branch underground.

Step 1: Constructing the ventilation network model

i) Building the network topology

Based on the data structure between branches and nodes in the ventilation network, construct the set of branches and the set of nodes, and create the undirected graph of the ventilation network. Construct a zero matrix, using branch resistance and branch air volume as weights, and assign values to the matrix by combining the set of branches and the set of nodes. This completes the initial setup of the branch resistance set and the air volume set for the ventilation network. Construct the column vector for the fan characteristics based on the fan characteristic equations.

ii) Minimum spanning tree calculation

Initialize parameters and use Kruskal algorithm to search for the minimum weight edges in the network, completing the calculation of the minimum spanning tree and the co-tree.

iii）Solving the fundamental loop matrix ***C***

Calculate the fundamental incidence matrix ***B*** of the ventilation network based on the relationships between nodes and branches; using the equation ***BC***^T^=**0**, solve for the fundamental loop matrix ***C*** of the network.

Step 2: Constructing the main function of the airflow regulation model

i) Initial condition setup

Set the decision variable independent optimization function *N*_2_ as the main function and the environmental variable independent optimization function *N*_1_ as the auxiliary function. Set the maximum number of iterations *T*_iter_. Set the iteration termination condition as when the correction value of the main optimization variable Δ*h_i_* is less than the iteration precision *σ*. keep the main function model in its default state.

ii) Spatiotemporal discretization

Use the stepwise difference method to discretize the mine roadways into finite micro-segments; use the environmental parameters at the end of the *i*-th segment as the starting environmental parameters for the (*i*+1)-th segment. Set the time variable *t* with an interval of 1 hour, ranging from 0 to 23, resulting in a total time interval of [0, 23] hours, discretized into time steps 1, 2, …, *t_u_*, …, 23.

iii) Function construction

Construct the main function *N*_2_ and the auxiliary solving function *N*_1_ based on the objective function decomposition equation (16). Obtain the branch density data *ρ*(*t_u_*) at each time step through model1. Calculate the fluctuating natural ventilation pressure *h*_N_(*t_u_*)′ and the ventilation resistance *h_i_*_-r_(*t_u_*)′ through model2. Determine the optimal solution for the airflow regulation model through model3.

iv) Function solving and output

Complete the optimal solution for airflow regulation and output the data for the adjustment branch set.

Step 3: Define the model1 for transient heat and airflow flow characteristics of airflow in time series

i) Fitting of state properties values for external mine nodes

Input atmospheric environmental field parameters to construct temperature, pressure, and density vectors. Utilize the polyfit function to complete polynomial fitting with *t* as the independent variable, obtaining *T*_1_(*t*)、*P*_1_(*t*) and *ρ*_1_(*t*).

ii) Construction of computational model for internal mine node state properties

Assign values to density, heat release coefficient, and heat conductivity matrices based on rock formation properties, and calculate the unsteady heat transfer coefficient. Establish a corresponding transient heat and airflow flow characteristics model for airflow according to the polytropic exponent-density-pressure-temperature coupling equation: *ρ=f*_3_(*n*, *P*, *T*, *t*).

iii) Calculation of state properties values

Set dynamically changing atmospheric node state parameters in time series as initial node values. Implement segmented recursive calculation of the transient model under discrete spatial scales.

Step 4: Define the model2 for fluctuating natural ventilation pressure and ventilation resistance in time series

i) Updating ventilation resistance weights

Reallocate the global ventilation network resistance weights based on the relationship *R_t_*_+1_=*R_t_*×*ρ_t_*_+1_/*ρ_t_*.

ii) Calculation of fluctuating natural ventilation pressure and ventilation resistance

Substitute *ρ_t_*_+1_ and *R_t_*_+1_ as initial values into the fluctuating natural ventilation pressure and ventilation resistance model. Compute the transient fluctuation values of natural ventilation pressure and ventilation resistance in time series, and output the natural ventilation pressure *h*_N_(*t_u_*)′ and ventilation resistance *h_i_*_-r_(*t_u_*)′ data at each time step.

iii) Optimizing airflow distribution

Use *h_i_*_-r_(*t_u_*)′ and *h*_N_(*t_u_*)′ at each time step as initial values in the ventilation network calculation model. Complete the calculation of the global airflow values at each moment, and output the airflow data *q_i_*(*t_u_*)′ at each time step.

Step 5: Solving the airflow regulation model3

i) Solving the main function

Transform the objective function and constraints into the standard form of linear programming. Construct the initial simplex tableau and check if the reduced costs meet the non-negativity condition to determine if the current solution is optimal. If the reduced costs are non-negative, end the iteration and output the current solution. If any reduced cost is negative, select the column corresponding to the most negative reduced cost to enter the basis. Calculate the ratios to determine the variable to leave the basis, and update all variable values centered around the pivot using Gaussian elimination to construct a new simplex tableau. Repeat the non-negativity check and variable replacement steps until the iteration conditions are met. Extract the optimal solution of the main function and the corresponding decision variable values from the final simplex tableau.

ii) Solving the auxiliary function

Update the ventilation resistance matrix based on the solution of the main function. Perform a secondary calculation of the ventilation network, generating airflow *q_i_*(*t_u_*)′′ and ventilation resistance *h_i_*_-r_(*t_u_*)′′ data for each time step after resistance adjustment. Use the corresponding airflow *q_i_*(*t_u_*)′′,, natural ventilation pressure *h*_N_(*t_u_*)′ , and ventilation resistance *h_i_*_-r_(*t_u_*)′′ as data sources at each time step. Calculate the instantaneous power consumption at each time step. Analyze the consumption trend of the system in time series based on the calculated *N*_1_ value. Solve for the minimum value min-*N*_1_ to complete the solution of the auxiliary function.

Q_4_: The term "frictional air resistance" should be changed to the total resistance of branches, equivalent to the resistance of branches in the real mine.

A_4_: Based on the law of turbulent frictional resistance, list the calculation equation of roadway ventilation resistance.

$$h_{i-\text{r}}=R_{i}q_{i}^{2}=\left(\alpha_{i}L_{i}U_{i}/S_{i}^{3}\right)q_{i}^{2}=\left(\lambda_{i}\rho_{i}L_{i}U_{i}/8S_{i}^{3}\right)q_{i}^{2}$$

Here, *h_i_*_-r_ is the ventilation resistance of roadway *i,* Pa; *R_i_* is the frictional resistance of roadway *i*, N∙s^2^/m^8^; *q_i_* is the airflow volume passing through roadway *i,* m^3^/s; *α_i_* is the friction coefficient of roadway *i,* kg∙m^3^; *L_i_* is the length of roadway *i,* m; *U_i_* is the perimeter of the cross-section of roadway *i*,m; *S_i_* is the cross-sectional area of roadway *i,*m^2^; *λ_i_* is the experimental proportional coefficient of roadway *i*; *ρ_i_* is the density of the airflow passing through roadway *i,* kg/m^3^.

Since English is not my native language, there may have been some misunderstandings of certain technical terms in my original text. The term "frictional air resistance" I previously used actually refers to *R* (N·s²/m⁸). In the field of mine ventilation, the more accurate term should be "frictional resistance." During the revision process, I consulted a glossary of professional terms in the mine ventilation field and confirmed that *R* represents "frictional resistance," while *α* represents the "friction coefficient." Additionally, I have referred to the resistance of mine branches as *h*_r_, meaning "ventilation resistance." I have checked and corrected all relevant terms throughout the article to ensure consistency in terminology.

Q_5_: Mine ventilation power is a strictly defined term in the science of mine ventilation. The authors probably use this term for a different purpose. Ventilation power and the electric power of engines are different parameters. It is proposed to define the term "mine ventilation" in the article.

A_5_: Your point about "ventilation power" and "electric power of engines" being different parameters is absolutely correct. In fact, in my earlier versions, I did not clearly distinguish between these terms. I mistakenly referred to all three (ventilation power, electric power of the engine, and system power consumption) as "mine ventilation power." This was indeed a misunderstanding on my part. What I intended to express was "system power consumption," not mine ventilation power. The objective function I constructed is also aimed at minimizing system power consumption. Therefore, I have reviewed and modified the relevant terms throughout the article to ensure consistent use of "system power consumption."

Q_6_: Similarly, "system power consumption" and "the flow power of the aerodynamic force" should be defined by appropriate equations so that the reader can easily understand the article.

A_6_: Regarding "system power consumption," it is first mentioned in the article because, when constructing the airflow regulation model based on the heat and airflow coupling characteristics, I considered fluctuating natural ventilation pressure and ventilation resistance as environmental variables, and adjustment resistance as the decision variable. The objective of airflow regulation is to minimize the power consumption of the ventilation system while meeting the airflow requirements of underground branches. Therefore, the definition of "system power consumption" can be directly reflected in the objective function (Section 2.3), ensuring that readers can clearly understand it.

$$\min\sum_{i=1}^{v} h_{i}\left(t\right)q_{i}\left(t\right)^{'}=\min\sum_{i=1}^{v} \left(\left(h_{i-\text{r}}\left(t\right)^{'}+\Delta h_{i}\left(t\right)+h_{\text{N}}\left(t\right)^{'}\right)\left(q_{i}\left(t\right)^{'}\right)\right)$$

As for "the flow power of the aerodynamic force," this was an inaccurate part of my expression. What I intended to describe was the force that drives the air flow. Due to my limited English proficiency, this might have caused misunderstandings. Therefore, I have removed such expressions to ensure more accurate communication and to avoid unnecessary ambiguities.

Q_7_: It is proposed that chapter 8 in McPherson's book in this manual be cited in the references.

A_7_: I referred to relevant content in McPherson's book "Subsurface Ventilation and Environmental Engineering," particularly Chapters 5, 8, 10 and 16. These chapters analyze the application of compressible fluids and thermodynamic effects in mine ventilation and delve into the energy conversion and transfer processes within mine ventilation systems. The theories in this book provided significant inspiration for my proposal of the natural ventilation pressure fluctuation model, helping me better understand the relationship between natural ventilation pressure fluctuations and thermodynamic processes in mine ventilation systems.

Following your suggestion, I have added "Subsurface Ventilation and Environmental Engineering" as a reference in the article and ensured proper citation to provide more comprehensive literature support.

Q_8_: In conclusion 1, the number of the developed equation should be given. The first sentence of Conclusion 2 needs to be simplified and should be rewritten more clearly and comprehensively to ensure the reader's full understanding. Conclusion 3 has not been proven to maintain a stable flow condition, and flow stability has yet to be defined.

A_8_: During the revision process, I found that the original conclusion had issues such as not comprehensively summarizing the research content and not describing the practical implications of the study. Therefore, I made substantial revisions to the conclusion. The revised conclusion is as follows:

Content Synthesis:

(1) To precise airflow control, three models are sequentially proposed: a transient heat and airflow flow characteristics model of airflow to quantitatively describe airflow state variations in time series; a fluctuating natural ventilation pressure and ventilation resistance model to quantitatively characterize their fluctuation patterns and impacts on ventilation systems; and an airflow regulation model to quantitatively determine optimal airflow regulation solution in time series.

Research Significance:

(2) The constructed time-series-based airflow regulation model incorporating heatand airflow coupling characteristic can accurately capture dynamic changes in airflow state properties and ventilation parameters. By real-time quantification of fluctuations in natural ventilation pressure and ventilation resistance caused by dynamic mine environmental characteristics, the model enables optimal real-time airflow regulation decisions at different time steps, achieving precise real-time ventilation network airflow regulation. This real-time precise airflow regulation reduces unnecessary air volume waste, thereby decreasing ventilation system power consumption and enhancing overall system economic efficiency.

Application Extension:

(3) Real-time precise airflow regulation dynamically responds to mine environmental changes, effectively improving the adaptive control accuracy and response speed of ventilation systems while optimizing control energy efficiency of the ventilation network. This approach provides crucial data support for the automation and intelligent development of mine intelligent ventilation systems, establishing a technical foundation for intelligent mine ventilation through dynamic regulation mechanisms and time-series-based optimization methods.

- **Other minor comments**

Q_1_: The term "stable airflow conditions: is used in the wrong context of the text.

A_1_: Due to my limited English proficiency, I misused the term "stable airflow conditions." In the original text, I intended to express "reducing system power consumption by 67.916 kW while ensuring stable airflow (without turbulence)," but I inadvertently used an inappropriate term, which caused misunderstandings. To address this issue, I have removed the related expression from the article and revised the content to ensure the accuracy and consistency of the terminology.

Q_2_: What does "state equation" mean when giving an approximation equation of the fan characteristic curve?

A_2_: This misunderstanding was actually caused by my inaccurate expression. In the original text, I intended to express "fan performance curve equation," but I mistakenly used the term "state equation," which led to confusion. In the approximate equation of the fan characteristic curve, I meant to describe the relationship between the fan performance and working conditions (such as airflow and pressure). Therefore, using "performance curve equation" would be more accurate. I have revised the article to ensure the consistency and accuracy of the terminology used.

Q_3_: The caption of Figure 7 needs to be corrected. The temperature color range is too extensive.

A_3_: Before conducting the simulation, I performed field measurements and calculations for parameters such as the length, area, perimeter, height difference, roadway temperature, and frictional resistance of the underground roadways. I also monitored and calculated the atmospheric pressure, atmospheric temperature, and the fan's pressure and airflow. Based on these data, I constructed the simulation model but did not mention the parameter values used in the model in the article. Therefore, I have rewritten the content of the numerical module, adjusted its structure, and added explanations of the mathematical model, physical model setup parameters, model solving methods, data collection methods, and analysis of simulation results to enable readers to assess the reliability and validity of the study.

Regarding the simulation result analysis module, I sampled the simulation results at the time points [0, 4, 14, 23] h, which are the extreme boundary points in time series, and plotted the dynamic temperature distribution cloud map under continuous spatiotemporal characteristics to analyze the simulation results (analysis of the interaction between airflow and the environmental field). During the analysis, I found significant changes in airflow state properties in roadways with height differences, while the state attribute values of airflow in horizontal roadways showed slight variations. Therefore, I used integral probes to collect data on the temperature field, pressure field, and density field of the model and listed the simulation data tables at [0, 4, 14, 23] h. By analyzing the specific value distribution of airflow state properties, I further analyzed the change patterns of airflow state properties.

During the analysis, I adjusted the temperature color scale range to [285, 310] K to better reflect the atmospheric environment field of the Gucheng Coal Mine and the significant fluctuations in airflow temperature at the inlet shaft in time series. This adjustment allows the temperature variation amplitude to more accurately match the actual environment.

I have supplemented and revised this part of the article, further optimizing the clarity and accuracy of the simulation results and charts to better explain the parameter selection and its impact on the simulation results.

Q_4_: It is recommended that the symbols used in the manuscript be standardized.

A_4_: Regarding the standardization of symbols, I have carefully reviewed the use of symbols in the article and ensured that the symbols are consistently expressed throughout the text. All symbols are defined upon their first appearance in the document and consistently used in subsequent sections to avoid any ambiguity or confusion. To further enhance the accuracy and standardization of the symbols, I also referred to standardized symbols and terminology from relevant fields to ensure that readers can more clearly understand the meanings of the models and equations.

Q_5_: English needs proofing.

A_5_: This is my first time writing an English paper, so there might be some language errors in my expressions. Indeed, during the writing process, I realized that my grasp of some professional terms and terminology was not precise enough, which might affect the fluency and accuracy of the article. In response, I have carefully reviewed the English expressions in the text, striving to make them more accurate and in line with academic standards. I will also continue to learn and improve my language skills in future research, especially in mastering professional terminology and expressions. Thank you for your suggestions; I will continue to improve and enhance the quality of my writing.
