# Supplementary material for: Decision on optimal airflow regulation solution set based on heat and airflow coupling characteristics of mine airflow in time series
Source: PLoS One. 2025 May 7;20(5):e0320326. doi: 10.1371/journal.pone.0320326 (PMC12058199; doi:10.1371/journal.pone.0320326)
Supplement: Attachment — Submitted filename: Reviewer comments.docx [file pone.0320326.s001.docx]

**Reviewer comments**

The manuscript addresses an important issue related to deep mining ventilation, particularly the mismatch between the ventilation power parameters and the underground ventilation network due to neglecting unsteady heat transfer between the airflow and the thermal environment. This mismatch makes it difficult to maintain the balance of airflow supply and demand in the mine roadways. The authors, based on the unsteady-state heat transfer leading to the fundamental nature of heat flow in airflow, propose a thermally corrected natural ventilation pressure formula and use this corrected pressure as an environmental variable to build a mathematical model for airflow control. By applying this model to the *Gucheng* coal mine, management decisions regarding airflow control in terms of ventilation power and resistance are made based on the model results. The research contributes to the optimization of intelligent ventilation and supplements the basic theory of the ventilation network under the heat-flow coupling characteristic of airflow, which is a novel approach. However, there are several parts in the manuscript that need to be addressed:

1. In Section 2.2, a numerical model of unsteady heat transfer for airflow in the *Gucheng* Coal Mine is presented. It is recommended to incorporate field measurement data or experimental data to validate the accuracy of the simulation model.

2. In Section 2.3, The proposed airflow control model, which is based on the heat-flow coupling characteristic of airflow, should be compared with currently commonly used control models. It is suggested that the proposed model be compared with commonly used control models to clearly explain the advantages and disadvantages of the new model.

3. In Section 3.1, there are errors in the notation of Equations 14 and 15; please review and correct them. In Section 3.2, the ventilation resistance calculation for the central ventilation network and *Taoyuan* ventilation network in Scheme 2.1 are incorrectly written; please check and amend accordingly.

4. Figures 8 and 10 represent the fan characteristic curves. The working conditions and blade angles should be clearly labeled in the figures to help readers observe the fan's status before and after adjustment.

5. The units throughout the paper should be consistent, and variables in the equations should be defined. The figures and textual descriptions should be as clear and understandable as possible. Some figures are unclear; it is advised that the author revise them to improve clarity.

6. In the conclusion section, while the main findings and contributions of the study should be clearly stated, the practical significance of these findings for the design, operation, and decision-making of intelligent ventilation systems should also be highlighted. The paper currently lacks a detailed discussion on the practical implications of the work, which should be addressed in the conclusion.

I hope the authors consider these points to improve the clarity and overall quality of the paper.
